# Missed Monteggia fracture in children: Is annular ligament reconstruction always required?

**DOI:** 10.4103/0019-5413.55978

**Published:** 2009

**Authors:** Atul Bhaskar

**Affiliations:** K J Somaiya Medical College, Paediatric Orthopaedic Surgeon, Bombay Hosptial Institute of Medical Sciences, Mumbai, India

**Keywords:** Missed Monteggia fracture, annular ligament reconstruction, ulna osteotomy

## Abstract

**Background:**

Chronic (neglected) radiocapitellar joint dislocation is one of the feared complications of Monteggia fractures especially when associated with subtle fracture of the ulna bone. Many treatment strategies have been described to manage chronic Monteggia fracture and the need for annular ligament reconstruction is not always clear. The purpose of this study is to highlight the management of missed Monteggia fracture with particular emphasis on utility of annular ligament reconstruction by comparing the two groups of patients.

**Materials and Methods:**

In a prospective study 12 patients with mean age of 7.4 years, who presented with neglected Monteggia fractures, were studied. All children underwent open reduction of the radiocapitellar joint. Five children (Group A) were treated with angulation-distraction osteotomy of ulna and annular ligament reconstruction and six cases (Group B) required only angulation-distraction osteotomy of ulna without ligament reconstruction. In one case an open reduction of the radiocapitellar joint was sufficient to reduce the radial head and this was included in Group B. The gap between injury and presentation was from 3 months to 18 months (mean 9 months). Ten patients were classified as Bado I, and one each as Bado II and III respectively. We used the Kim's criteria to score our results.

**Result:**

The mean follow-up period was 22 months. All ulna osteotomies healed uneventfully. The mean loss of pronation was 15 degree in Group A and 10 degree in Group B. Elbow flexion improved from the preoperative range and no child complained of pain, deformity and restriction of activity. The elbow score was excellent in 10 cases, and good in two cases.

**Conclusion:**

Distraction-angulation osteotomy of the ulna suffices in most cases of missed monteggia fracture and the need for annular ligament reconstruction is based on intraoperative findings of radial head instability.

## INTRODUCTION

Delayed diagnosis is a known complication observed after acute Monteggia fracture and its equivalent. This usually occurs if there is lapse in diagnosis or if the injury films have been inadequately taken. A late or missed Monteggia lesion is defined as one presenting four weeks after the initial surgery.^1^,^2^

Indications for surgery in children with chronic monteggia fracture are unclear. A child rarely gets pain and the functional limitations are minimal. Progressive valgus deformity of the elbow, limitation of forearm rotation, and pain with strenuous activity are some of the reported indications for surgery.^3^,^4^

Management of chronic monteggia injury poses several challenges. The difficulty of obtaining a satisfactory function is directly proportional to the duration of “missed dislocation”. This is because the surgical strategies required to achieve an anatomical reduction become proportionally complex as duration of the “missed dislocation” increases.^5^,^6^

Several treatment options are available to manage this difficult problem: open reduction of radiocapitellar joint is almost always required and some form of ulna osteotomy is needed to maintain the radial head in position. There is no clear consensus on requirement of annular ligament reconstruction to stabilize the radial head. Some authors have done ligament reconstruction in every case whereas others have reported its use only when there is residual instability of the radial head after ulna osteotomy.^7^,^8^ Annular ligament reconstruction is not without complications and hence its use in every case may not be required.^4^,^9^–^13^

Recent reports on the use of external fixation, with gradual lengthening and angulation of ulna osteotomy, leading to radial head reduction without even the need to expose the radiocapitellar joint^14^–^16^ suggest that annular ligament reconstruction is not always required to achieve stability of the radial head. No single treatment will fit in managing every case of missed Monteggia fracture and a rationale approach is recommended on a case to case basis.^17^

The purpose of this study is to highlight the management of missed Monteggia fracture with particular emphasis on utility of annular ligament reconstruction by comparing the two groups of patients.

## MATERIALS AND METHODS

Twelve cases of chronic monteggia fracture treated in the period 2002–08 were reviewed prospectively in the Children's Orthopedic Clinic, Mumbai. The mean age was 7.4 years (4 – 12 years) with seven boys and five girls in the study. The mean duration from initial injury to presentation, i.e., “missed dislocation” was nine months (3 months – 18 months). Ten cases were Bado^18^ type I injury where the radial head had dislocated anteriorly; one was type III where there was lateral dislocation of the radial head and one case (Case 5) which was treated by external fixation of the ulna that progressed to nonunion with posterior angulation of ulna and posterior dislocation of the radial head, i.e., type II pattern. Details of the 12 patients are given in Table 1.

**Table 1:** Clinical details of patients with missed monteggia fracture

Case	Age Yr/mths	Gender	Bado type	D^a^	Group	Signs	Pain	FU^b^ (mths)
1	7.2	B	I	8	A	Ant prominence	Nil	22
2	6.3	G	I	7	A	Ant prominence	Nil	21
3	7.4	B	I	18	A(BG)	Ant prominence	Nil	23
4	6.2	G	I	9	A	Nil	Nil	25
5	8.9	G	II	6	A(BG)	Ulna nonunion	Pain	14
6	4.3	B	I	3	B	Ant prominence	Nil	8
7	6.4	G	III	8	B	15° valgus elbow	Pain	4
8	5.6	B	I	14	B	15° valgus elbow	Pain	26
9	8.7	B	I	9	B	Nil	Nil	24
10	6.9	B	I	9	B	Ant prominence	Nil	26
11	12.2	B	I	10	B(BG)	Ant prominence	Nil	25
12	9.3	G	I	7	B	Ant prominence	Nil	26

Da = Duration of missed dislocation, i.e., time since injury, Group (Treatment Group): A = Annular ligament reconstruction performed, B = Annular ligament reconstruction not performed B = Boy, G = Girl,

FUb = Follow up period in months, BG = Bone grafting done, ant = anterior

In all cases except one (case 5) the ulna fracture had united. Of the 12 cases, in nine, the diagnosis of monteggia fracture was missed and patients were treated for ulna fracture. Two had plastic deformation of ulna which was unrecognized and one had undergone external fixation of ulna in an attempt to reduce the radial head by distraction (case 5).

Elbow function was assessed for activity pain, range of motion, deformity and function.

For the study, informed consent was sought from parents and the patient records were available for review.

At initial presentation, only three patients complained of pain. Seven children were dissatisfied with their elbow appearance and complained of “popping like sensation” due to anterior prominence of the radial head. Terminal 10-20° of flexion was restricted in seven cases and two cases with pain had progressive elbow valgus. Two children had flexion contracture of 10°. One patient had complete loss of supination and in four cases there was terminal restriction of 10°. Pronation was mildly restricted in three cases. The remaining four children exhibited motion comparable to opposite elbow. Details are given in Table 2.

**Table 2:** Elbow function in patients

Case		Range of motion					
		
	Type/Surgery	Normal unaffected elbow treatment group		Affected elbow			
				
				Pre-op	Post-op	Pre-op	Post-op
						
		flex/n/ext	pro/n/sup	flex/n/ext	flex/n/ext	pro/n/sup	pro/n/sup
1	A: Ulna plating	140-0-5	90-0-80	140-0-5	130-0-0	90-0-80	80-0-80
2	A: K-wire fixation of ulna	135-0-5	90-0-80	120-0-5	130-0-5	90-0-80	70-0-80
3	A: Ulna plating + BG	130-0-5	90-0-90	120-0-0	130-0-0	80-0-70	70-0-70
4	A: Ulna plating	140-0-0	90-0-80	130-0-5	110-0-0	90-0-90	70-0-80
5	A: Ulna plating + BG	140-0-5	80-0-90	60-20 (FFD)	120-10 (FFD)	80-0-0	70-0-40
6	B: Only open reduction done	130-0-0	80-0-90	130-0-5	130-0-5	90-0-90	80-0-90
7	B: K-wire fixation of ulna	140-0-5	90-0-90	140-0-5	130-0-0	90-0-80	80-0-80
8	B: Ulna plating	130-0-0	80-0-90	130-10 (FFD)	140-0-0	80-0-90	80-0-80
9	B: Ulna plating	140-0-5	90-0-90	130-10 (FFD)	140-0-5 (FFD)	90-0-80	80-0-90
10	B: K-wire fixation of ulna	135-0-5	80-0-90	130-0-5	120-0-0	90-0-80	90-0-80
11	B: Ulna plating + BG	135-0-5	90-0-80	120-0-0	130-0-0	90-0-90	80-0-80
12	B: Ulna plating	140-0-5	80-0-90	140-0-5	130-0-0	80-0-90	80-0-80

FFD = Fixed flexion deformity, flex/n/ext: Full flexion/neutral/full extension, pro/n/sup: Pronation/neutral/supination, K–wire = Kirschner wire fixation of ulna, BG = Autogenous bone graft used

In one case an external fixator was applied after ulna osteotomy to attempt a closed reduction of the radial head. However, this technique failed and the patient presented with painful nonunion of the ulna, shortening and deformity of forearm and gross limitation of elbow motion (20° – 60° of flexion was present). This patient had complete loss of supination. No child had any neurovascular deficit.

All patients underwent surgery using the posterolateral approach described by Boyd,^19^ where a single incision was made to expose the radiocapitellar joint and ulna bone. An open reduction of the radiocapitellar joint was performed in all cases and an ulna angulation-distraction osteotomy was performed in 11 cases. Five children who underwent annular ligament reconstruction were classified as Group A [Figure 1] and seven cases where no ligament reconstruction was performed were classified into Group B [Figure 2]. The radiocapitellar joint was first approached and all fibrous tissue and remnants of annular ligament were excised to facilitate reduction of the radial head. Although the radial head appeared enlarged there were no gross dysplastic changes in radial head and capitellum. In one case (Case 6), simple excision of the scar tissue was sufficient and no ulna osteotomy was required to maintain radiocapitellar stability. In nine cases an ulna apex-posterior osteotomy was performed at the site of previous deformity or in the proximal third of ulna and in one type III dislocation case an apex-medial osteotomy was performed. The osteotomy site was fixed with a K-wire or plate depending on the size of the bone. The degree of angulation and distraction required to maintain the radial head in position was determined intraoperatively. The osteotomy site was provisionally fixed with a Kirschner wire or a plate with two screws, one proximal and one distal to the osteotomy and radiocapitellar stability was assessed in pronation and supination. The radiocapitellar joint is most stable in full supination and usually slips out with pronation depending on the inclination and shortening of the ulna. If the radiocapitellar joint was unstable in pronation and did not remain stable, even after changing ulna angulation and distraction, an annular ligament reconstruction was performed to augment the radial head stability. This was required in five cases. In six cases adjustment of the ulna angulation and fixation sufficed to stabilize the radial head. For annular ligament reconstruction the lateral slip of the triceps fascia was harvested and its distal attachment to the ulna was preserved. This was then passed around the radial neck and sutured to itself and the periosteum of the proximal ulna. In group A, the ulna osteotomy was fixed with 3.5 reconstruction plate in four cases and with a single Kirschner wire in one case.

**Figure 1A:**
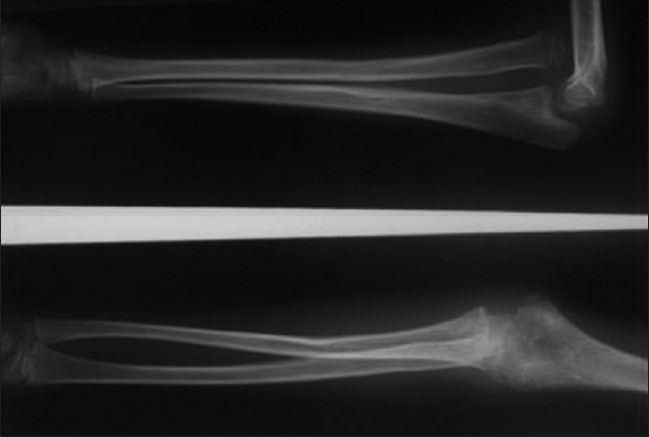
Lateral and anteroposterior radiograph of right elbow of a seven year old girl showing missed Monteggia fracture of 18 months

**Figure 1B:**
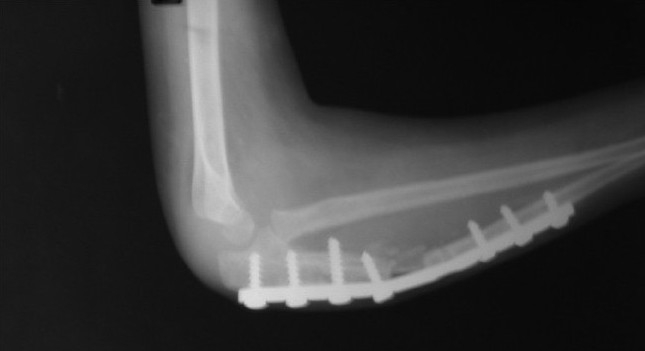
Lateral radiograph of elbow and forearm after open reduction, ulna osteotomy, bone grafing and annular ligament reconstruction

**Figure 1C:**
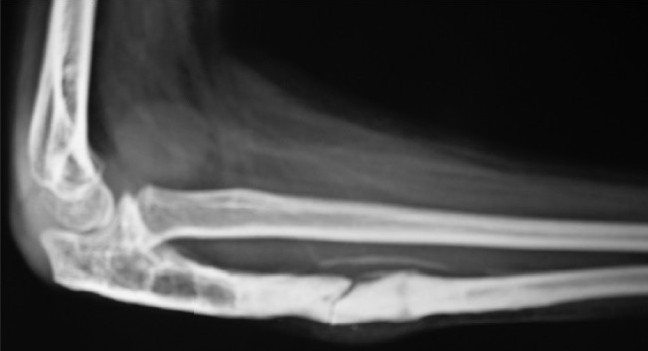
Lateral radiograph of elbow at final follow-up of 24 months. She had no symptoms and the graft has healed well. The radial head is well maintained

**Figure 2A:**
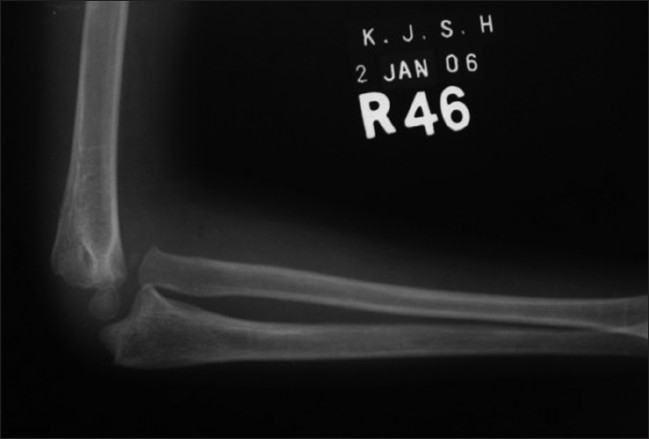
Lateral radiograph of elbow of 12 year old child showing missed Monteggia fracture

**Figure 2B:**
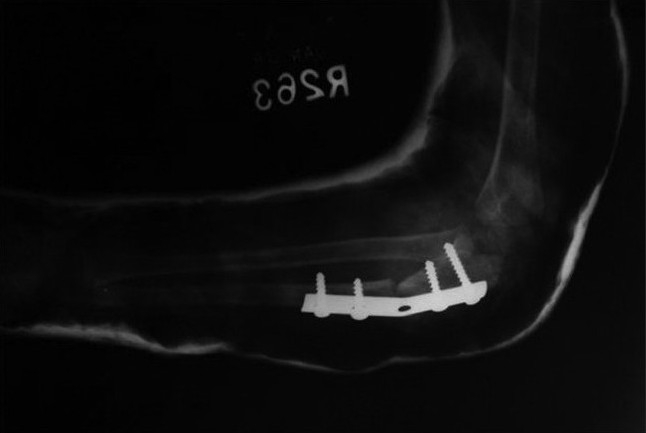
Post-operative lateral radiograph of the elbow showing the angulation-distraction osteotomy of the ulna with bone grafting. Annular ligament reconstruction was not required (Group B)

The ulna plate was applied on the dorsal surface of the bone and in two cases (Cases 3 and 5) a temporary transcapitellar pin was inserted intraoperatively to assess the optimal alignment of the ulna before its fixation. This pin was removed once the osteotomy was fixed. In no case was a transcapitellar wire left insitu and no case required a radius osteotomy.

In two cases (Case 3 and 11) where the osteotomy distraction gap was greater than 10mm, the residual gap was filled with autograft taken from the iliac crest. The case with ulna gap nonunion underwent plating and bone grafting too (Case 5). Ulna angulation and length restoration was all that was required to maintain reduction of the radiocapitellar joint in six cases. In group B, wo osteotomies were fixed with Kirschner wires and a 3.5 mm reconstruction plate was used in four cases [Table 2].

Postoperatively all patients had their elbows immobilized in an above elbow cast which was kept for 8 – 10 weeks with the elbow in 90° flexion and full supination. After cast removal, patients were referred for both active and passive physiotherapy.

Radiographs were taken after cast removal and at follow-up to detect any residual subluxation and healing of the ulna osteotomy. All children were reviewed at four weekly intervals after cast removal for initial assessment of motion for three months; then at three- month intervals to assess the final function.

The mean follow up period was 22 months (8 – 26 months). At the last follow-up, all patients were rated according to the functional elbow score devised by Kim^8^. The elbow was assessed for deformity, pain, range-of-motion and function. The four parameters were weighted equally, 25 points each, for a perfect score of 100 points: 1) deformity: 25, no concern; 15, minor concern; 0, major concern; 2) pain: 25, no pain; 15, intermittent mild pain but not limiting activities; 0, pain, limiting activities; 3) range of motion (sum of the flexion-extension and pronation-supination arcs): 25, greater than 250°; 15, 250°–200°; 0, less than 200°; 4) function: five activities of daily living (comb hair, feed self, open door knob, hold on to subway overhead rail, put on shoes with hands) were identified and were given a weight of five points each if the patient could perform such tasks without a problem. If the patient could not accomplish these tasks, a zero was given for each task he or she failed to perform without difficulty. Total elbow performance score was graded as excellent (90 or more points), good (89–75 points); fair (74–60 points); or poor (less than 60 points).

## RESULTS

The mean follow-up period was 22 months (8 – 26 months). At the last follow-up, all patients were rated according to the functional elbow score devised by Kim^8^.

The mean age in both the groups, A and B, was 6.8 years and 7.1 years and the mean duration of “missed dislocation” was 9.6 months and 8.5 months respectively.

The mean healing time for the ulna osteotomy for all children was eight weeks (6 – 14 weeks). The elbow range of motion was recorded for all patients.

The mean loss of elbow flexion was comparable in groups, 10° in Group A and B respectively. The mean loss of pronation was 15° and 10° in Group A and B respectively. Supination was mildly restricted in three cases (less than 10°). One child in Group A (case 4) had re subluxation of the radial head which did not require any treatment. One child in Group B had superficial wound infection which resolved with local dressing. No infection was seen in Group A.

Comparing the flexion-extension and pronation-supination arc in both groups revealed that for Group A, excluding the case of nonunion (case 5) the mean flexion arc reduced by 20°. In group B there was a mean gain of 10°. The pronation-supination arc reduced in both groups (excluding case 5) with the mean loss of pronation of 15° and 10° in groups A and B respectively. The fixed flexion deformity (FFD) in one patient (case 8) completely corrected and in one case (case 9), terminal FFD of five degree's was present but this didn't compromise the result. Case 5 in group A had gross restriction of movement pre-operatively but improved considerably after surgery. The flexion arc improved from 40° to 110° and the pronation-supination arc improved from 80° to 130° [Table 3].

**Table 3:** Details of pre and postoperative elbow function

Case No	Flexion arc (in degrees)			Change	Pain	Pro/Supination arc (in degrees)			Kim's score
					
	Normal side	Pre-op	Post-op			Pre-op	Post-op	Change	
1	145	145	130	-15	Nil	170	160	-10	Excellent
2	140	125	135	10	Nil	170	150	-20	Excellent
3	135	120	130	10	Nil	150	140	-10	Excellent
4	140	135	110	-25	Nil	180	150	-30	Excellent
5	145	40	110	70	Nil	80	130	50	Good
6	130	135	135	0	Nil	180	170	-10	Excellent
7	145	145	130	-15	Nil	170	160	-10	Excellent
8	130	120	140	20	Nil	170	160	-10	Excellent
9	145	120	145	25	Nil	170	170	0	Excellent
10	140	135	120	-15	Nil	170	170	0	Excellent
11	140	120	130	10	Nil	180	160	-20	Good
12	145	145	130	-15	Nil	170	160	-10	Excellent

Negative value indicates loss of motion

No child complained of activity related pain or disability in daily function. No child complained of “popping sensation” experienced earlier. The carrying angle was restored to normal 10^°^ in the two cases with elbow valgus (cases 7 and 8).

Radiographs revealed the osteotomy site had healed well by 8 weeks and there was good incorporation of bone graft used in two cases in Group A and one in Group B. One child had 2mm residual subluxation of the radial head (case 4).

At mean follow up of 22 months, the ulna angulation osteotomy had gradually remodeled in five cases without causing any radial head disturbance. Kim's criteria [Table 4] was used to score the elbow postoperatively with the result: 10 elbows reported excellent outcome, and two had a good result (Case 5 and 11). Case 5 had total motion less than 250° and case 11 had some residual tenderness but no activity related pain. In view of the small sample size, no comparisons were made between the groups. In three children plates were removed after one year and no refracture was reported.

**Table 4:** Kim's criteria

Criteria			
**Deformity**	No concern	Minor concern	Major concern
**Score**	25	15	0
**Pain**	No pain	Mild – Intermittent	Activity limiting
**Score**	25	15	0
ROM*	> 250°	200-250°	< 200°
**Score**	25	15	
**Function**	No problem	With difficulty	Unable
ADL^	25	15	0

ROM* - Sum of flexion-extension and pronation-supination arc, ADL^ - Activities of daily Living: Five functions assessed: Comb hair, Feed self, Open door knob, Hold overhead, Put shoes

## DISCUSSION

Treatment of missed monteggia lesion presenting for four weeks poses several challenges. The probability of obtaining closed reduction of the radial head at that stage is almost negligible and some form of surgery is required to restore normal anatomy.^2^,^8^,^12^ However, the reason for surgery is not always clear. Many children are asymptomatic and only present because of the deformity. The other issue is the duration of the “missed dislocation” which precludes a good result. Authors have reported successful reconstruction as late as four years after injury.^20^–^23^ Left untreated, the children adapt well to the anomalous joint position in missed monteggia fracture, but advancing age can compromise the result of surgery.^24^ There are a few reports of surgery on children over 10 years with good functional results.^8^,^24^,^25^

In this series the oldest child was 12 years and the longest interval between injury and treatment was 18 months. Both the children had an excellent outcome. The interval of missed dislocation in the older child was 10 months and probably did not result in significant dysplastic changes in the radiocapitellar joint.

The type of reconstruction varies and there is no clear consensus regarding treatment of missed monteggia fracture. Some authors have reported that open reduction of the radial head is sufficient and ulnar osteotomy is not required.^25^,^26^ Others have reiterated the need for ulna osteotomy, almost always, to restore radial head alignment.^23^,^26^,^27^ Although simple ulna osteotomies without fixation have been described,^28^ the ulna angulation obtained after radial head reduction necessitates internal fixation to prevent re displacement of the radial head.^29^

The role of annular ligament reconstruction in maintaining radial head reduction has never been critically analyzed. Some authors have advocated its use in every case that requires open surgery on the radiocapitellar joint. Reconstruction involves harvesting a fascial slip from the triceps aponeurosis or the forearm fascia and creating a loop around the radial neck. Boyd^22^ used a slip from the extensor aponeurosis; Bell-Tawse^9^ used the central slip of triceps fascia and Lloyd-Roberts^10^ modified this using the lateral slip attached distally. In theory this fascial slip acts both as a dynamic and static stabilizer and prevents radial head subluxation. Stoll *et al*,^2^ described eight cases of missed monteggia fracture treated with annular ligament reconstruction; the radiocapitellar joint was transfixed with Kirschner wire in two cases to enhance the stability.

We had five cases in which ligament reconstruction was required to maintain the radial head reduction. In six cases no ligament reconstruction was performed. The mean age, duration of dislocation and type of lesion were comparable in both the groups. The decision to perform annular ligament reconstruction was based on intraoperative stability of the radiocapitellar joint obtained after fixation of the ulna osteotomy. Often slight distraction with posterior angulation of the ulna would enhance the anatomical alignment of the radiocapitellar joint. Three cases in this series required bone grafting. One case for nonunion and the other two to enhance stability of radial head reduction.

The child with missed lesion for 18 months required annular ligament reconstruction to stabilize the radial head and also ulna distraction-angulation ostetomy with bone grafting. One child in group B required angulation-distraction with bone grafting. The duration of missed dislocation was 11 months. Perhaps, the key to adequate radial head reduction is the technique of ulna osteotomy and only a large multicenter series or meta analysis can throw some more light on this intriguing problem.

The precise angulation and distraction would vary in each case but future biomechanical studies on this subject should clarify the relationship between ulna angulation and enhanced radiocapitellar stability.^30^

Some authors have used gradual ulna distraction with external fixator to effect radial head reduction without even opening the joint.^14^,^16^ These reports highlight the importance of ulna lengthening and angulation in maintaining stability of the radial head. Hasler^20^ reported 15 cases of missed monteggia fracture in which external fixation of the ulna osteotomy was combined with open reduction of the radiocapitellar joint.^29^ No patient underwent annular ligament reconstruction and no re displacement of the radial head was reported at a mean follow up of 22 months in his study. Inoue and Shionaya stressed the importance of ulna angulation as three of their six patients with simple osteotomy without angulation had persistent dislocation of the radial head.^27^

Annular ligament reconstruction is not without complications. Gyr and Stevens reported on 15 children who underwent annular ligament reconstruction.^11^ All children had some limitation of forearm rotation and four cases had asymptomatic radial head re subluxation. They emphasized on ligament reconstruction to prevent the need for radial head excision in future. After ligament reconstruction, restriction of forearm rotation has been reported by several authors, as also nerve injury, myositis ossificans and re-displacement of the radial head are some of the documented complications.^31^–^33^

In this study, all children in group A and B had limitations of forearm rotation. Pronation was more limited and this could perhaps be related to the position of forearm immobilization in supination. However, the limitation of elbow motion did not affect any function.

In conclusion, this study stresses the importance of alignment of the ulna in restoring radial head stability and that annular ligament reconstruction is not always necessary. It would be prudent to quote a line from Campbell's textbook of Orthopedics, “regardless of how little or how much remodeling has taken place, an osteotomy is usually necessary to lengthen the ulna and produce a stable radial head reduction.”
